# Interactions between the Nicotinic and Endocannabinoid Receptors at the Plasma Membrane

**DOI:** 10.3390/membranes12080812

**Published:** 2022-08-22

**Authors:** Ana Sofía Vallés, Francisco J. Barrantes

**Affiliations:** 1 Instituto de Investigaciones Bioquímicas de Bahía Blanca (UNS-CONICET), Bahía Blanca 8000, Argentina; 2 Laboratory of Molecular Neurobiology, Institute of Biomedical Research (BIOMED), UCA-CONICET, Av. Alicia Moreau de Justo 1600, Buenos Aires C1107AFF, Argentina

**Keywords:** plasma membrane, membrane domains, nanodomains, neurotransmitter receptors, cannabinoids, acetylcholine receptor, cannabinoid receptor

## Abstract

Compartmentalization, together with transbilayer and lateral asymmetries, provide the structural foundation for functional specializations at the cell surface, including the active role of the lipid microenvironment in the modulation of membrane-bound proteins. The chemical synapse, the site where neurotransmitter-coded signals are decoded by neurotransmitter receptors, adds another layer of complexity to the plasma membrane architectural intricacy, mainly due to the need to accommodate a sizeable number of molecules in a minute subcellular compartment with dimensions barely reaching the micrometer. In this review, we discuss how nature has developed suitable adjustments to accommodate different types of membrane-bound receptors and scaffolding proteins via membrane microdomains, and how this “effort-sharing” mechanism has evolved to optimize crosstalk, separation, or coupling, where/when appropriate. We focus on a fast ligand-gated neurotransmitter receptor, the nicotinic acetylcholine receptor, and a second-messenger G-protein coupled receptor, the cannabinoid receptor, as a paradigmatic example.

## 1. Introduction

Current studies on biological membranes support the notion that they constitute highly specialized structures with compartmentalized functional regions of varying thickness, lipid composition, and functional properties. Signaling pathways require a highly regulated membrane organization since these processes are characteristically dynamic and occur in separated spatio-temporal discrete domains. Of great relevance to cell physiology are the lateral membrane heterogeneities enriched in cholesterol, sphingolipids, and phospholipids with saturated fatty acid acyl chains. Their chemical composition determines their engrossed thickness and their physicochemical properties are akin to those of the liquid-ordered (Lo) domains observed in artificial membranes. These membrane regions, also known as lipid rafts [1,2], play a crucial role in manifold cellular processes by providing the structural substrate for functional compartmentalization. Distinct cellular signaling mechanisms, in particular those operating at the cell-surface membrane, often rely on these discrete lateral domains to perform in an isolated manner in close but separate locations of the membrane. Interactions between domains harboring different signaling proteins can result in the dynamic regulation or modulation of these signaling mechanisms in their synergic potentiation, or their silencing. One example of such an interacting signaling system is discussed in this short review: the endocannabinoid (EC) system and the nicotinic acetylcholine receptors (nAChRs).

The EC system comprises a wide variety of lipid-signaling molecules, enzymes, and receptors. Endocannabinoids (ECs) are important homeostatic modulators of neuronal activity in the central nervous system (CNS). nAChRs are a Cys-loop gene family of neurotransmitter receptors, belonging to the superfamily of pentameric ligand-gated cation channels (pLGIC). In the mammalian brain, there is ample combinatorial diversity of nAChR subtypes that allows a diversity of functional responses to the endogenous neurotransmitter, acetylcholine (ACh), and to a much broader spectrum of chemical compounds that modulate this receptor, such as positive and negative allosteric modulators (PAMs and NAMs, respectively), general anesthetics, fatty acids, and cholesterol, to mention the most important ones.

ECs and EC receptors anatomically and functionally interact with nAChRs in brain areas such as the midbrain, the hippocampus, and the amygdala [3,4,5]. ECs regulate cholinergic afferents on the ventral tegmental area (VTA) dopaminergic neuronal cells, and therefore play an important role in modulating reward stimuli and addiction. Indeed, through multiple interactions nAChR and EC receptors crosstalk in the CNS and induce neuroadaptations in response to diverse stimuli. All these molecular processes including the down or upregulation of selective nAChR subtypes, the modulation of neurotransmitter release, and neuronal scaffolding proteins take place within or in the proximity of the plasma membrane.

In this review, we focus on the heterogeneous composition of the plasma membrane and the significance of compartmentalization for the efficiency of synaptic signaling involving nAChRs and their modulation by the EC system. In addition, we discuss the structural and functional roles played by lipids and proteins in such membrane nanodomains, and their relationship with the EC system and the nAChRs.

## 2. The Nanodomains within the Plasma Membrane

### 2.1. Cholesterol

The neutral lipid cholesterol plays a key role in membrane compartmentalization [6,7] given that its content and topographical distribution are main determinants of the membrane’s physicochemical properties. Cholesterol increases membrane rigidity and thickness and alters tension within the membrane [8,9,10,11,12]. Thus, cholesterol affects the morphology of the plasma membrane. The cholesterol molecule has a small polar headgroup (-OH) and a hydrophobic region (rigid sterol ring) that changes the physicochemical properties of phospholipid bilayers. Higher lateral pressure (tension) is exerted at the headgroup of the cholesterol molecule, while lower lateral pressure takes place at the hydrophobic regions of the molecule. Cholesterol-containing membranes provide a negative spontaneous curvature which is counterbalanced by the extension of acyl chains of phospholipids that thicken the hydrophobic region of the bilayer, thus compensating, at least in part, for this curvature and reducing lateral pressure (or curvature). Furthermore, the hydrophobic region of the cholesterol molecule restricts the flexible fitting of adjacent protein regions. Cholesterol can induce pressure on transmembrane proteins due to changes in the lateral pressure profile, which varies along the depth of the bilayer [13].

Cholesterol can diffuse in these liquid-ordered (Lo) lipid domains, albeit with slower diffusion rates than in liquid-disordered (Ld), cholesterol-poor regions, where cholesterol can diffuse at the rate of submicroseconds [14]. Cholesterol molecules can also flip-flop between the outer and inner leaflets of the membrane bilayer. Cholesterol distribution within biological membranes is influenced by the affinity of sterol for other lipids and proteins present in the plasmalemma. For instance, cholesterol-phosphatidylcholine and cholesterol-phosphatidylethanolamine associations are common at the inner layer of liquid-ordered domains, whereas the outer layer harbors sphingomyelin-cholesterol associations, thus providing two different liquid phases with different diffusion rates [15,16,17].

Cholesterol interacts with several neurotransmitter receptors. These transmembrane molecules share cholesterol-consensus linear binding sequences, such as the so-called cholesterol recognition/interaction amino acid consensus motifs (CRAC) and its mirror image, CARC [18]. These consensus domains facilitate the incorporation of many membrane proteins into cholesterol-rich domains [19]. Cholesterol was experimentally shown to modulate various pentameric ligand-gated ion channels (pLGICs) [20,21], as well as members of the superfamily of G-protein coupled receptors (GPCRs) [22]. The muscle-type nAChR exhibits a cholesterol-recognition motif (“CRAC”) adjacent to the transmembrane helix M1, and another cholesterol-recognition sequence of opposite orientation (“CARC”) on the M4-facing surface of M1, adjacent to one of the proposed cholesterol-binding cavities [18]. Similarly, the transmembrane helix 7 of human cannabinoid receptor 1 (CB1R) displays a CRAC sequence [23].

Cholesterol interacts with the actin subcortical cytoskeletal meshwork contributing to receptor clustering and compartmentalization [6,24]. The cognitive decline that has been described upon ageing is associated with irreversible loss of membrane cholesterol [25,26]; replenishment of cholesterol in hippocampal slices from aged mice was found to improve learning and memory in those affected [26].

Together with cholesterol, sphingomyelin contributes to the membrane-actin cytoskeleton crosstalk by modulating membrane binding and the activity of the Rho GTPases, a family of small signaling G proteins and subfamily of the Ras superfamily. Sphingomyelin accumulation leads to a decrease in metabotropic glutamate receptors and F-actin content in a Niemann–Pick disease type A mouse model, defective in acid sphingomyelinase, precluding the membrane attachment of RhoA and its effectors ROCK and profilin IIa [27].

### 2.2. The Complex Array of Proteins in the Synaptic Plasma Membrane

Resident proteins and interacting lipids within the plasma membrane dynamically orchestrate many signaling cascades. Alterations of the membrane lipid composition can result in defective functions of raft-associated proteins, and consequently favor abnormal cell signaling. For example, abnormal cholesterol metabolism has been implicated in the development of neurological disorders such as Alzheimer and Parkinson diseases, Huntington disease, Niemann–Pick type C disease, and schizophrenia spectrum disorders [28,29,30,31,32]. Likewise, alteration of membrane-associated proteins can also hamper many important cell functions and even lead to pathological states. In Alzheimer and Parkinson diseases, loss of plasma membrane integrity in neuronal cells is often observed. This condition favors toxic amyloid-β and α-synuclein aggregation [33,34,35] through lipid-induced conformational changes [34,36,37,38] or mass action [39,40] that, when combined, increase the probability of inter-molecular interactions and promote aggregation [39,40]. During aggregation, amyloid-β oligomers extract phospholipids from the plasma membrane and incorporate these lipid molecules into the growing fibrils, thus causing membrane rupture. Disease proteins can increase membrane permeability by different mechanisms [40], such as protein-induced membrane rigidity [41,42], membrane thinning [43,44] and deformation [43,44,45,46], as well as detergent-like effects [38,41,47], and pore formation [36,37,42,48,49,50]. These pores in the bilayer result in Ca^2+^ influx from the extracellular compartment and efflux of cytosolic content.

Many proteins at the synapse dynamically modulate synaptic activity, thus influencing a great variety of biochemical states and protein–protein interactions that control protein synthesis and contribute to the reorganization of cytoskeletal architecture [51], which is essential for adequate dendritic spine morphology [52]. For instance, actin filaments can associate with actin-binding proteins (ABPs) to provide the necessary structure to modulate membrane morphology [53]. However, the actin cytoskeleton and its regulatory proteins are not merely structural proteins: they also act as scaffolds for stably positioning and anchoring other scaffolding proteins, ion-channels, neurotransmitter receptors, cell-adhesion proteins, and other macromolecules at the cell surface that contribute to the architectural integrity and function of the postsynaptic density (PSD) [54]. They are also necessary for the trafficking of subcellular structures such as endosomes, and for higher brain functions such as memory formation and extinction [55]. At the PSD, the family of PDZ domain-containing scaffold proteins offers a molecular interface between glutamatergic receptors (GluRs) and the cytoskeleton [56,57]. For example, two proteins that contain PDZ domains, PSD95 and SHANK (SH3 and multiple ankyrin repeat domains), can bind through multiple interactions to NMDA receptors and to the metabotropic glutamate receptor (mGluR) [58,59]. The postsynaptic expression of SHANK was shown to enhance presynaptic function [59], suggesting a key role for the SHANK scaffold in synaptic plasticity.

Another group of proteins that are tethered to membranes by lipid groups attached to their C-termini are the Rab proteins. These proteins belong to the Ras superfamily of small GTPases. They are predominantly present in an active state, whereas the dephosphorylation of GTP by hydrolysis to GDP converts Rab proteins to their inactive form. Rab proteins have been implicated in the facilitation of membrane-associated receptor transport. Some key Rab proteins identified as participating in the internalization and recycling of membrane receptors are Rab 5, Rab 4, and Rab 11. The former is located on the cytoplasmic surfaces of the plasma membrane and is specifically associated with the internalization of clathrin-coated pits and with the fusion of early endosomes [60,61]. Rab 4 is generally described to facilitate the rapid recycling of receptors directly from endosomes and targeted back to the plasma membrane [62,63]. Rab 11 is associated with the slow recycling of GPCRs via the perinuclear recycling compartment [64]. Thus, the regulation of the number and the availability of receptors at the plasma membrane strongly depends on Rab proteins.

## 3. Endocannabinoids

### 3.1. The Endocannabinoid System

ECs are involved in a plethora of physiological and pathological processes in mammalian cells, having effects on mood, appetite, reproduction, immunity, memory, and pain perception [65]. The EC system comprises cannabinoid receptors (CBRs), endogenous cannabinoid ligands (endocannabinoids, Ecs), and the enzymes involved in their synthesis and degradation. Ecs are lipid molecules, synthesized de novo by phospholipase action after hydrolyzing the lipid precursors from the cellular membrane [66]. The Ecs anandamide (N-arachidonoylethanolamine, (AN)) and 2-arachidonoylglycerol (2-AG) are lipid messenger molecules. AN is an endogenous lipid neurotransmitter derived from the polyunsaturated fatty acid arachidonic acid and a member of the N-acylethanolamines (NAEs) family [67]. It is a partial agonist of cannabinoid receptor 1 (CB1R) and cannabinoid receptor 2 (CB2R), and a full agonist of the vanilloid receptor 1 (VR1), whereas 2-AG acts as a full agonist of CB1R and CB2R. Additionally, ECs can activate other “non-CB” receptors, such as the G-protein-coupled receptor 55 (GPR55) and peroxisome proliferator-activated receptors (PPARs) [68,69,70,71].

CB1Rs are expressed at very high levels in a subset of GABAergic interneurons, such as the cholecystokinin (CCK)-containing basket cells in the forebrain [72], and at lower levels on many glutamatergic terminals throughout the brain [73]. Within neurons, quantitative electron microscopy studies revealed that CB1Rs are found mainly on the pre-terminal axonal segment and less on more proximal axons, dendrites, or the cell soma [74].

### 3.2. The Endocannabinoid System in Synaptic Transmission

**Figure 1:**
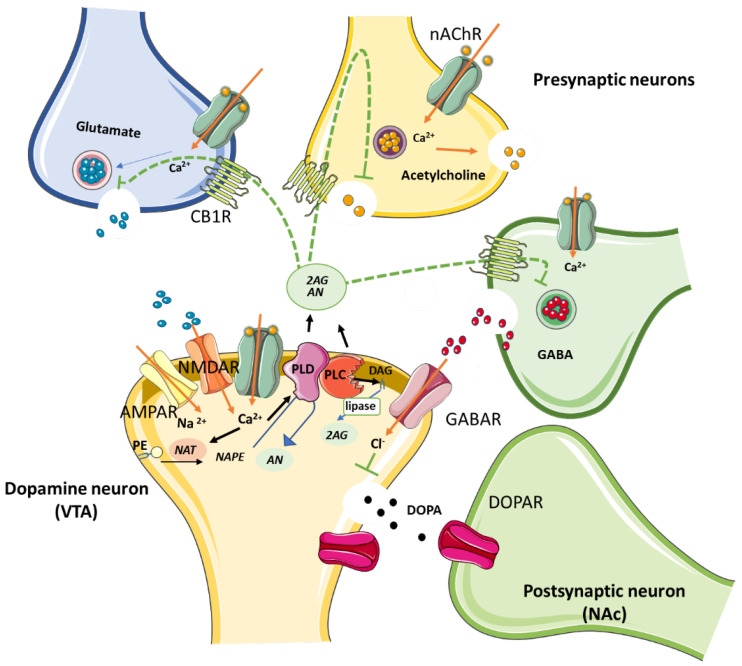
Schematic illustration of the biosynthetic pathway followed by ECs N-acylethanolamine (AN) and 2-arachidonoylglycerol (2-AG) by NAPE-PLD and DAG lipase, respectively. Both AN and 2-AG bind to presynaptic CB1Rs expressed on GABAergic, glutamatergic and cholinergic terminals, precluding neurotransmitter release. Induction of DOPA secretion by primary inhibition of GABA release is involved in the reward pathway in the brain. Abbreviations: PLD, N-acyl phosphatidylethanolamine phospholipase D; DAG, diacylglycerol; NAT, N-acyltransferase; PE, Phosphatidylethanolamine; nAChRs, nicotinic acetylcholine receptors; CB1R, cannabinoid type 1 receptor; AMPA, amino-3-hydroxy-5-methyl-4-isoazolepropionate receptor; GABA, γ-aminobutyric acid; GABAR, GABA receptor; NMDAR, N-methyl-D-aspartate receptor; DOPA, dopamine; DOPAR, DOPA receptor; VTA, ventral tegmental area; NAc, nucleus accumbens.

In contrast to classical neurotransmitters, ECs are produced as needed [75]. Upon postsynaptic depolarization, for example, after activation of metabotropic glutamate, muscarinic, or dopamine D2 receptors [76,77,78], the synthesis of ECs takes place through Ca^2+^ influx and the activation of the Gq-protein, or via phospholipase C and D activation (Figure 1). However, AN and 2-AG have different synthetic and metabolic pathways [79]. While the former is mainly synthesized from N-acyl-phosphatidylethanolamine (NAPE) by NAPE-specific phospholipase D (NAPE-PLD) and metabolized by fatty acid amidohydrolase (FAAH) [79], the latter is predominantly synthesized from 2-arachidonoyl-containing phospholipids by DAG lipase (DAGL) and metabolized by monoacylglycerol lipase (MAGL). Once synthesized, ECs are released into the synaptic cleft and are able to activate CBRs in presynaptic and/or nearby GABAergic, glutamatergic, and cholinergic neurons [77,80,81,82,83].

The latter retrograde EC modulation of GABA terminals enables the depolarization-induced suppression of inhibition (DSI) through a transient suppression of GABA released onto the postsynaptic neuron, thereby disinhibiting it. EC action on CBRs located on the glutamate terminal, on the other hand, favors a depolarization-induced suppression of excitation (DSE) [84,85] by inhibiting glutamate release, hence activating postsynaptic neurons. However, the role of DSI is reported to predominate over that of DSE due to differences in CB1R sensitivity between inhibitory and excitatory synapses [86]. Importantly, the predominant GABA suppression in the midbrain is suggested to be the mechanism that induces dopamine (DA) release at mesocorticolimbic and nigrostriatal sites [87] associated with reward-based learning and also with addiction [88,89].

The EC system has a central role in the modulation of synaptic transmission throughout the CNS. ECs regulate behavior through reward learning, by modulating mesolimbic reward circuits that include the ventral tegmental area (VTA), the nucleus accumbens (NAc), and the lateral habenula (LHb). It is these CNS functions that have given ECs notoriety in relation to drug abuse and addiction behaviors. Furthermore, EC-like lipid-signaling molecules are increasingly understood as neuromodulators able to regulate dopaminergic transmission, as well as reward and addiction behavior. Thus, upon interacting with different receptors within the plasma membrane, ECs stimulate a great variety of signaling pathways. As we will see, membrane microdomains are key players in modulating signal transduction through the organization of CBRs.

Other nonspecific mechanisms of action, e.g., involving the alteration of the host lipid bilayer, have been suggested for ECs [90]. AN, for instance, was shown to reduce the ionic current of many voltage-gated ion channels in the presence of antagonists of CB1Rs and CB2Rs [91,92,93,94], thus suggesting a receptor-independent mechanism for the modulation of multiple membrane protein functions.

### 3.3. The Endocannabinoid System Organization in Membrane Microdomains

**Figure 2:**
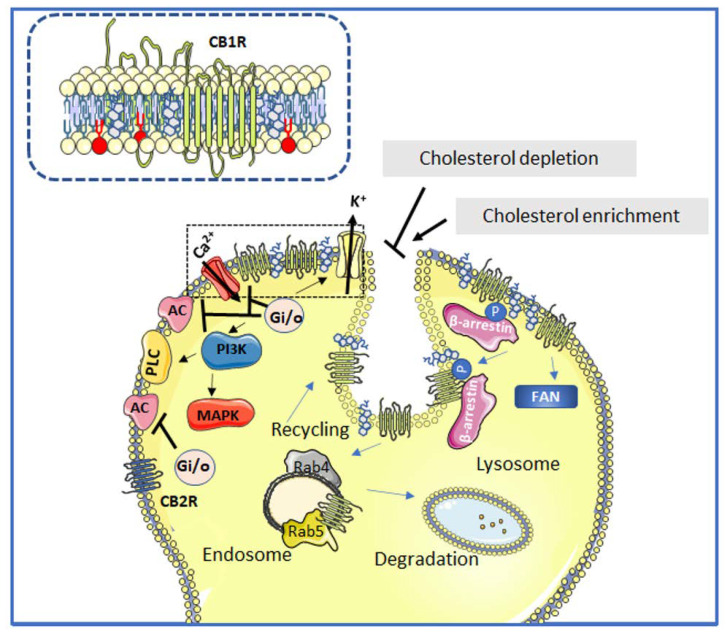
Schematic depiction of the interactions between cannabinoid receptor signaling and lipid domains. Neuromodulation upon agonist binding to CB1Rs and CB2Rs (unphosphorylated receptors) induces Gi/o-dependent inhibition of adenylyl cyclase (AC) activity and activation of different MAPK cascades. CB1R positively regulates inwardly rectifying K^+^ channels, whereas it negatively regulates voltage-gated Ca^2+^ channels. In addition, the CB1R can activate PLC at the plasma membrane and can also signal through non-G proteins such as the adaptor protein FAN. The phosphorylated CB1R is a target of β-arrestin. The attenuation of CB1R signaling at cholesterol-rich lipid domains occurs through a Rab5-dependent endocytic mechanism. Once internalized, the CB1R may either be recycled back to the plasma membrane through a Rab4-dependent mechanism or further targeted to lysosomal degradation. The CB2R does not reside in or interact with Lo lipid platforms. Alteration of ordered lipid domain integrity either by depleting or supplying cholesterol is shown to inhibit or enhance the internalization of CB1R, respectively. The *inset* depicts a closer view of the cholesterol-rich Lo lipid domain (marked with a dotted line in the main figure). Cholesterol molecule in gray, sphingolipids in red and other phospholipids in yellow.

Once activated by ECs, either CB1R or CB2R trigger a signaling cascade through the inhibitory Gi and Go subtypes of the G proteins (i.e., Gai1, Gai2, Gai3, Gao1, Gao2) [95,96]. Their activation by ECs leads, in turn, to the inhibition of adenylyl cyclase, the activation of mitogen-activated protein kinases, the inhibition of certain voltage-gated calcium channels [97,98], and the activation of G protein-linked inwardly rectifying K+ channels [96,99,100] (Figure 2).

As shown in Figure 1 and Figure 2, the CB1R can also modulate ion channel proteins [96]. The effects of CBR activation are long lasting because of the several secondary messenger molecules involved in processes that play major roles in neuronal plasticity [101,102]. This makes apparent the diversity of signaling pathways that can be elicited by different CB1R and CB2R agonists. However, the great capacity of CBRs to couple to different subtypes of G proteins does not make them less specific in triggering downstream signaling transduction mechanisms. The plasma membrane plays a major role in confining cannabinoid responses, both spatially and temporally. Cholesterol/sphingomyelin-rich domains provide CBRs with dynamic and organized platforms where assembly of signaling complexes can take place and, in addition, prevent crosstalk between different pathways [103]. Reinforcing the relevance of membrane domains for the EC system signal selectivity, CB1R binding and signaling are demonstrated to be influenced by membrane compartmentalization [103].

Chemicals such as methyl-β-cyclodextrin deplete cholesterol from the plasma membrane (Figure 2) and prevent the onset of cell death by completely blocking the ability of AN to induce superoxide generation, phosphatidylserine exposure, and p38 MAPK activation. However, the use of a CBR antagonist did not prevent cell death in primary hepatic stellate cells [104], thus suggesting a more complex CBR–lipid interface interaction that is disturbed upon cholesterol depletion. In agreement with this observation, the activation of CB1R-dependent adenylate cyclase signaling by AN is reported to be almost doubled by methyl- β-cyclodextrin treatment, pointing to the relevance of these lipid platforms in cell signaling processes [103]. Conversely, upon membrane cholesterol enrichment, CB1R-dependent signaling was shown to be reduced by half in primary cultures and immortalized cell lines [105,106].

CB1Rs are physically associated with ordered lipid domains in a cholesterol-dependent manner (Figure 2). Cholesterol depletion alters AN-induced CB1R endocytosis and its subsequent trafficking to the lysosomal compartment, pointing to the importance of lipid platforms in the intracellular trafficking of the CB1R [107]. No such information is available on CB2Rs. Lipid platforms can physically sequester different signaling components, preventing crosstalk between different pathways [108], or favor internalization of CB1Rs via caveolae-related endocytosis (Figure 2), negatively regulating CB1R function [109]. For example, the desensitization process of CB1R involves phosphorylation of specific serine residues in the intracellular loop III and C terminal regions. Phosphorylation of these serine residues is performed by protein kinase C (PKC) [110] and G-protein-coupled receptor kinase 3 (GRK3) [111], promoting the recruitment of β-arrestin 2 [111]. These three molecules (PKC, GRK3, and β-arrestin 2) are either resident at or dependent on Lo lipid domains [112], which may explain why, upon disruption of these domains, the desensitization process of CB1R fails and signaling through the CB1R is sustained. Once phosphorylated by PKC and GRK3, β-arrestin 2 is recruited and CB1R is targeted for internalization towards either low pH endosomes and subsequent recycling back to the cell surface, or to late endosomes and lysosomes for degradation (Figure 2). The former endocytic and recycling cycle of the CB1R is facilitated by the small GTPases Rab5 and Rab4, respectively (Figure 2) [62].

Although CB1R is present at the plasma membrane, in HEK-293 cells approximately 85% of the heterologously expressed receptors are localized in intracellular vesicles [62], suggesting a predominantly intracellular localization at a steady state. In agreement with these observations, the intracellular localization of CB1R was previously described in AtT20 cells [113] and hippocampal neurons [114]. Intracellularly located CB1Rs cannot interact with ECs or exogenous cannabinoids, thus preventing stimulation. These data support CB1Rs being constitutively endocytosed. Lo lipid domain instability associated with the diminution of cholesterol content at the plasma membrane would lead to a reduction of the endocytic process and an increase in the number of CB1Rs at the plasma membrane available for ligand interaction. This explains why cholesterol depletion from plasma membranes can favor an enhanced cellular response to ECs, by augmenting the availability of such receptors.

CB2R is also regulated by the Rab family. Specifically, Rab 5 has been implicated, as is also the case with CB1R, in the modulation of the endocytic internalization, whereas Rab11 is reported to participate in a slower recycling process via the perinuclear recycling compartment [64].

Agonist-CBR interactions also mediate lipid raft dynamics. If CB1R are activated in a transient manner, sphingomyelin breakdown is initiated, and ceramides accumulate through functional coupling with the adaptor protein FAN [115]. Conversely, if CB1R are continuously stimulated, G protein-dependent de novo ceramide synthesis takes place through activation of serine palmitoyl transferase activity [116].

### 3.4. Endocannabinoid Interactions with Endocannabinoid Receptors within the Plasma Membrane

The plasma membrane plays a major role in the interaction between AN and CBRs. In order to deliver its biological message, after being synthesized, AN leaves the postsynaptic membrane, crosses the synaptic cleft, and finds its way to CB1Rs at the presynapse. AN is an amphipathic derivative of arachidonic acid with a polar head of an ethanolamine group. It is considered a lipid-derived neurotransmitter molecule with enhanced water solubility. Because of its lipidic nature, however, it is very unlikely that AN interacts with CB1Rs via their extracellular region; the plasma membrane on the other hand provides an adequate microenvironment for AN to interact with the transmembrane helices of the receptor [117,118]. For this to occur, AN has to firstly penetrate the lipid bilayer and, secondly, diffuse in the plasmalemma to find its binding site [117,119]. Cholesterol again is a central player in the process of guiding AN towards its receptor. Cholesterol by itself suffices to act as the AN transporter at the plasma membrane [120]. AN is deemed to exhibit a specificity for cholesterol in biological membranes over other lipids [121]. This interaction is thought to occur through a hydrogen bond between the –OH group of the sterol and the –NH group of AN [121] that, once established, enables cholesterol to trigger the insertion of AN to the plasma membrane through a flip-flop mechanism favoring the development of van der Waals interactions that stabilize the complex [121]. It should be noted that this process is unlikely to occur within Lo lipid domains, where cholesterol is tightly packed with sphingomyelin [122,123] and is hence inaccessible to AN; it is more likely to occur in non-raft, liquid-disordered domains of the plasma membrane with greater accessibility to AN. Since CB1Rs in the brain are mostly found in Lo lipid domains [107,124], to reach its binding site, AN must penetrate these domains either by passive diffusion (owing to its lipid nature) or by a membrane transport system. Again, cholesterol seems to be the molecule that performs these transport and delivery functions.

From an energetic point of view, AN has a much higher affinity for CB1R than for cholesterol (energy of interaction ~ −136 kJ mol^−1^ vs. −30.3 kJ mol^−1^) [125]. This thermodynamic characteristic has led to the suggestion that, when the AN-cholesterol complex reaches the CB1R, AN detaches from cholesterol and moves towards the receptor. The AN binding site on the CB1R has been localized between the transmembrane 6 (TMH6) and 7 (TMH7) regions of the GPCR [121]. TMH7 of the CB1R carries a CARC domain [23,121] that is available for cholesterol interaction. It is proposed that cholesterol could attract AN back, contributing to the release of AN from the binding site, and enabling the CB1R to revert to the unbound state after receptor activation. In addition, the cholesterol-AN complex leaves the polar head of the neurotransmitter fully accessible for enzymatic hydrolysis by FAAH [121]. All in all, the plasma membrane provides the EC system with multiple compartments to regulate the synthesis, transport, and even degradation of its neurotransmitters. It harbors metabolic enzymes and further contributes to the enhancement or abolishment of signal transduction.

## 4. nAChR

nAChRs are excitatory, cationic pLGICs that reside in presynaptic, postsynaptic, and extrasynaptic membranes in the central and peripheral nervous system [126,127,128,129,130]. They are essential players in neurotransmission across different types of synapses and in muscle contraction. nAChRs can be activated by their natural neurotransmitter, ACh), or by a wide variety of ligands [131,132]. nAChRs at the plasma membrane can adopt multiple conformational states. Upon agonist exposure, the closed state of the channel becomes activated and in the “open state”, the influx of small cations can take place. After depolarization of the cell, the channel can adopt a desensitized state that is unable to be activated by ligand binding or return to a closed state.

Seventeen different subunits (α1–α10, β1–β4, γ, ε, and δ) encoded by seventeen genes in vertebrates that combine and form either homo- or hetero-pentameric structures, have been described [133]. The muscle-type nAChR found at the neuromuscular junction is formed from four distinct subunit types organized in an (α)_2_βγδ pentamer, the fetal γ-subunit being replaced by the ε-subunit in the adult. The subunit composition of the nAChR determines the kinetics of the conformational stages of the channel. For example, while α4β2 nAChRs desensitize rapidly when exposed to the agonist nicotine, α7 nAChRs do not desensitize as fast [134,135,136]. Also, the subunit composition affects the selective cationic permeability of the nAChR and the pharmacological affinities of various agonists [137,138,139,140].

nAChRs influence synaptic plasticity by increasing intracellular Ca^2+^ release, inducing long-term potentiation (LTP) and favoring a depolarization state. The activation of presynaptically located nAChRs results in the release of many neurotransmitters including dopamine, norepinephrine, GABA, and Glu in a Ca^2+^-dependent manner. Heteromeric α4β2 and homomeric α7 nAChRs are the most abundant neuronal subtypes [141,142]. The activation of these receptors in hippocampal interneurons indirectly affects neurotransmitters release (Glu or GABA) by activating voltage gated calcium channels [143,144]. Furthermore, presynaptic α4β2 nAChR activation by the agonist nicotine is reported to induce dendritic spine enlargement as a consequence of increased Glu concentration and of glutamatergic neurotransmission [145], thus affecting synaptic plasticity. Activation of other neuronal nAChRs, such as the α3β4 nAChR, at presynaptic sites is shown to stimulate tetrodotoxin-insensitive GABA release via T-type voltage gated calcium channels and Ca^2+^ from internal stores [146].

At postsynaptic sites, the activation of nAChRs also produces significant inward (depolarizing) currents in neurons in many brain regions and modulates synaptic plasticity. A7 nAChR at postsynaptic sites can regulate glutamate receptors and, hence, glutamatergic signaling [147,148], as well as GABAergic interneuron activity [149,150]. Halff and coworkers proposed a mechanism by which α7 nAChRs would modulate synaptic potentiation independently of fast excitatory transmission [7]. They report that the activation of α7 nAChRs at postsynaptic sites recruits GluA1 receptors from the surface pool of mobile extrasynaptic receptors, this in turn contributing to stabilization of GluA1 receptors in the neuronal spine and producing an increase in the density of this subtype of glutamatergic receptors. However, the participation of functional PSD-95 scaffold protein family members is required to anchor GluA1 macromolecules at the postsynaptic membrane. Both activation and inhibition of α7 nAChRs in the prelimbic cortex result in the induction of LTP [151]. Modulating network excitability by cholinergic signaling must therefore be finely regulated for adequate neural excitability and plasticity to take place.

### 4.1. nAChR Localization within the Plasma Membrane

The neuronal nAChRs are found in brain areas considered to be involved in learning, cognition, and memory, such as the basal forebrain, hippocampus, cerebellum, and the temporal cortex [152]. nAChRs are found within lipid platforms in the plasma membrane. These cholesterol/sphingolipid-rich lipid domains determine nAChR nanocluster topography function and mobility on cell surfaces [153,154]. Numerous papers over the past 40 years have provided data on the multiplicity of effects of cholesterol on the peripheral nAChRs found in skeletal muscle, and in the electromotor synapse of electric fish.

nAChRs are influenced by cholesterol concentration at various levels of organization, within multiple time windows, and during ontogenetic development and adulthood [20,155]. Cholesterol homeostasis dysregulation produces alterations in the biophysical properties of the membrane bilayer that affect the mobility and protein–protein interactions of neurotransmitter receptors [22].

Cholesterol/sphingolipid/ceramide-rich lipid platforms are required for both muscle-type and neuronal nAChR trafficking to the plasma membrane [153,156,157,158,159]. Indeed, our group has provided evidence that disruption of these lipid platforms leads to both altered nAChR function and cell-surface expression [153,154].

Lowering cholesterol levels at the plasma membrane causes rapid nAChR (muscle type) internalization, and compensatory gain-of-function of the nAChRs remaining at the plasma membrane [160,161,162]. Studies using stimulated emission depletion (STED) [163] and single-molecule localization (SMLM) [164,165] superresolution microscopies further show contrasting changes in the distribution of nAChR nanoclusters and individual molecules upon cholesterol depletion or enrichment. In addition, changes in the cholesterol concentration of the membrane followed using fluorescence recovery after photobleaching and fluorescence correlation spectroscopy [153] and SMLM [164,165] have also been shown to modify the translational mobility of the receptor in the plane of the plasma membrane. Likewise, cholesterol content in the cell-surface membrane modulates neuronal nAChR subtypes. In a recent study by Báez-Pagán and coworkers, a reduction in the macroscopic response of the neuronal α7 nAChR subtype was described as cholesterol to phospholipid ratios increased [166]. The α7 nAChR subtype of nAChRs is predominantly located in cholesterol-rich Lo domains [157,159,167,168]. Other studies from our laboratory indicate that long-term inhibition of cholesterol biosynthesis can differentially augment cell-surface levels of both α4β2 and α7 nAChRs in the neurites and soma of rat hippocampal neurons [169].

Bearing in mind that neuronal nAChRs can be found on both the soma and synaptic terminals of excitatory and inhibitory neurons in different brain regions (Figure 1), the lipid microenvironment of the plasma membrane where they reside plays a crucial role in the modulation of nAChR responses. Additionally, alteration of cholesterol content at the plasma membrane will affect neuronal excitability through the modulation of the nAChR channels and by indirectly impacting on GABAergic and glutamatergic transmission, in turn possibly impinging on multivariable physiological or pathophysiological outcomes.

### 4.2. nAChR and EC Receptor Crosstalk

The overlapping distribution of CBRs and nAChRs is not fortuitous. Their distribution anatomically overlaps in brain areas such as the midbrain, the hippocampus, and the amygdala. Many papers support the notion that nicotinic and EC systems interact bidirectionally in the brain reward pathway [170,171,172,173,174,175,176,177,178,179,180,181,182,183,184,185,186,187]. Buczynski and coworkers, reported that nicotine self-administration in rats modified AN levels in the VTA [188]. Furthermore, fluctuations in AN but not 2-AG are associated with withdrawal from nicotine [189]. The lipophilic nature of ECs allows their incorporation into the plasma membrane, and their presence is shown to modulate the action of alcohol and volatile anesthetics on α7 nAChRs [190,191], suggesting that an alteration of the physicochemical properties of the plasma membrane is taking place. Using *Xenopus* oocytes, Oz and coworkers demonstrated that AN can reversibly inhibit nicotine-induced currents in a concentration-dependent manner without significantly affecting its half maximal effective concentration (EC50) value. These authors suggest that AN behaves as a noncompetitive antagonist of α7 nAChRs [192,193]. Likewise, AN was shown to inhibit the peak amplitudes of α4β2 nAChR-mediated currents in SH-EP1 cells [194] and in myenteric neurons [92,93]. In addition, synthetic cannabinoids have been reported to modulate cholinergic neurotransmission in the hippocampus [170,185,186,187]. In the latter, nAChRs and CB1Rs play a role in the cognition-impairing effects of the main psychoactive ingredient of cannabis, δ-9-tetrahydrocannabinol (THC) [195]. In the mesolimbic dopamine system, they are implicated in brain reward processes [195,196,197]. The blockade of α7 nAChR by methyllycaconitine at non-toxic concentrations reduces the behavioral and neurochemical effects of THC related to its abuse [198]. It has therefore been suggested that drugs that block α7 nAChR could be useful agents in the treatment of cannabis abuse in humans [198]. Contrarily, THC is reported to increase the response of the α7 nAChR to ACh by 128% [199]. Cannabidiol (CBD), the other most abundant component of cannabis, is reported to reduce cigarette consumption by 40% and to reduce the response of the α7 nAChR to ACh by 49% [199]. Understanding the complex interactions between the cholinergic and the endocannabinoid systems in the nervous system is critical to increasing our chances of using cannabinoids in the clinic as therapeutic tools.

Cohen and coworkers reported that SR141716 (rimonabant), a CB1R antagonist, blocks the motivational and dopamine-releasing effects of nicotine in rats [200], while other authors have also observed that rimonabant decreases the motivation to self-administer nicotine [201]. In support of the existence of a physiological interaction between the cannabinoid and cholinergic systems, genetic deletion of CB1R in knockout mice was demonstrated to inhibit nicotine-induced rewarding effects, evaluated by a conditioned place preference paradigm (CPP) [202,203]. In addition, a blockade of the CB1R in various areas of the brain, such as in the shell of the NAc, the basolateral amygdala, the prelimbic cortex, and in the bed nucleus of the stria terminalis, was reported to produce a reduction in nicotine-seeking behavior [204,205]. Genetic deletion or pharmacological inhibition of FAAH is shown to enhance the expression of nicotine CPP [203], thus suggesting that ECs play an important role in the rewarding properties of nicotine. Furthermore, CB1R stimulation in rats was reported to increase the motivation to self-administer nicotine and to enhance cue-induced reinstatement of nicotine-seeking behavior [206]. In contrast, neither the activation nor the inhibition of CB2R was shown to produce effects on the motivation to obtain nicotine or nicotine intake in rats [207]. However, studies performed in mice have documented the relevance of CB2R receptors on the rewarding/reinforcing properties of nicotine [208,209]. Thus, the current literature supports a distinct profile of CB1R and CB2R effects on behavior, and suggests that there may be important species differences mediating these effects.

The modulatory crossover effects taking place between CBRs and nAChRs at the plasma membrane point to EC and cholinergic systems as holding significant promise for the development of novel therapeutic strategies in behavior and addiction.

## 5. Concluding Remarks

Biological membranes are much more complex than double-layered lipid barriers acting simply as a boundary between the intracellular content and the extracellular medium. They are highly organized and multifaceted structures that provide an asymmetrical interface where many transport and enzymatic processes can simultaneously occur. The direct observation of nanoscopic lateral heterogeneities in the plane of the membrane accomplished in the last two decades has provided structural grounds that help explain how these dynamic platforms control and govern signal transduction, neurotransmitter synthesis, metabolism, and degradation. Here, we briefly reviewed the importance of these supramolecular structures and mechanisms using the CB1R and the nAChR as an example of two interacting cell-surface signaling systems. We also stressed the importance of the neutral lipid cholesterol in the phenomena involving these paradigmatic receptors. Cholesterol plays a direct role in orchestrating the membrane organization and endocytosis of the two receptor systems, contributes to the transport of the lipidic endocannabinoid AN to and from the CB1R, and participates in the regulation of the number, distribution, and functional states of nAChRs.
